# Supplementary material for: Neutral competition explains the clonal composition of neural organoids
Source: PLoS Comput Biol. 2024 Apr 22;20(4):e1012054. doi: 10.1371/journal.pcbi.1012054 (PMC11065252; doi:10.1371/journal.pcbi.1012054)
Supplement: S1 Supplemental Methods — (PDF) [file pcbi.1012054.s001.pdf]

S1 Supplemental Methods

for "Neutral competition explains the clonal composition of neural organoids"

Florian G. Pflug, Simon Haendeler, Christopher Esk, Dominik Lindenhofer, Jürgen A. Knoblich, Arndt von Haeseler

Relative linage size frequencies

To plot the empirical lineage size frequencies (Figure 1B, Figure S3) we first assigned each lineage size a density by numerical differentiation of the empirical CDF (after conversion of the ECDF from a piece-wise constant to a piece-wise linear function). The resulting density was then smoothed using loess regression with degree 2 and smooth parameter 0.75 as implemented by R's loess function.

Exponential growth of the size and decay of the number of fast-growing lineages

A mathematically simple example of a model that predicts lineage sizes to follow a truncated Zipfian law with index α is that of exponential grow (with rate γ) of fast-growing lineages together with an exponential decay (with rate $\sigma = \alpha\gamma$) of the *number* of fast-growing lineages. Under this model, the time τ at which a lineage leaves the fast-growing regime is exponentially distributed with density $f(\tau) = \sigma e^{-\sigma\tau}$. Conditional on this time τ, the size of a lineage at time t is $s(\tau) = e^{\gamma \min\{\tau,t\}}$. To find the unconditional distribution of lineage sizes at time t, we consider $s(\tau)$ to be a change of variables from τ. Since $s(\tau)$ is invertible for all lineages sizes up to $e^{\gamma t}$, applying the change of variables formula yields the lineage size density

$$\frac{d\tau}{ds} \cdot f\left(s^{-1}(s)\right) = \frac{1}{s\gamma} \cdot \sigma \cdot e^{-\frac{\sigma}{\gamma}\log(s)} = \alpha s^{-1-\alpha}$$

where $\alpha = \sigma/\gamma$ and provided that $s < e^{\gamma t}$. Lineage sizes thus follow a Zipfian distribution with index α, truncated at size $e^{\gamma t}$.

Perturbation Simulations

We simulated three perturbed versions of the SAN model where we modified the rates of division and/or differentiation starting with day 11. (A) A reduced rate (one half, and one tenth) of asymmetric division and therefore a reduced output of N-cells per A-cell. This can be interpreted as a model of reduced neurogenesis by precursor cells such as RGCs. (B) Reduced rate of S-cell division and differentiation while keeping the net

growth rate (division minus differentiation) intact. This can be interpreted as a simple model of an ASPM knockout. (C) No symmetric divisions post day 11, rate of asymmetric division as large as is biologically realistic (2 divisions per day) and no final differentiation of A into N cells (rate of A to N differentiation is zero). Even though the N-cell output of A-cells is maximized, the lack of symmetric division reduces the maximal linages sizes by an order of magnitude.

Replicate Experiments

Lineage tracing of cerebral organoids was performed as outlined in Esk *et al.* [1]. Briefly, two retroviral libraries based on pRSF retrovirus expressing TagBFP were used for lineage tracing, which contain one of two semi-random barcode libraries. hESC cells were infected at low MOI to ensure largely single virus / barcode integration. Single cell suspensions were prepared, sorted for TagBFP and plated in Essential 8 media supplemented with RevitaCell to recover for 1-2 days before the organoid protocol was initiated. Organoids were harvested on days 1, 6, 11 and 42, 3 replicates for each day, and genomic DNA extracted by phenol/chloroform extraction. After 12 freeze/thaw cycles viral barcode inserts were amplified by using lineage tracing genomic DNA primers, which also introduced sample indices and sequencing adaptors. Resulting libraries were sequenced on Illumina HiSeq 2500 and Novaseq S1 lanes. As described by Esk *et al.* [1], samples were demultiplexed, LID error correction was performed by the UMI-tools directional algorithm, and LIDs that occurred in multiple samples were removed in samples with less than 95% max read count. Additionally, LIDs with low read count were removed according to a sample-specific threshold.

References

1. Esk C, Lindenhofer D, Haendeler S, Wester RA, Pflug F, Schroeder B, et al. A human tissue screen identifies a regulator of ER secretion as a brain-size determinant. Science. 2020;370: 935–941.
