# Supplementary figures and images for: Neutral competition explains the clonal composition of neural organoids
Source: PLoS Comput Biol. 2024 Apr 22;20(4):e1012054. doi: 10.1371/journal.pcbi.1012054 (PMC11065252; doi:10.1371/journal.pcbi.1012054)

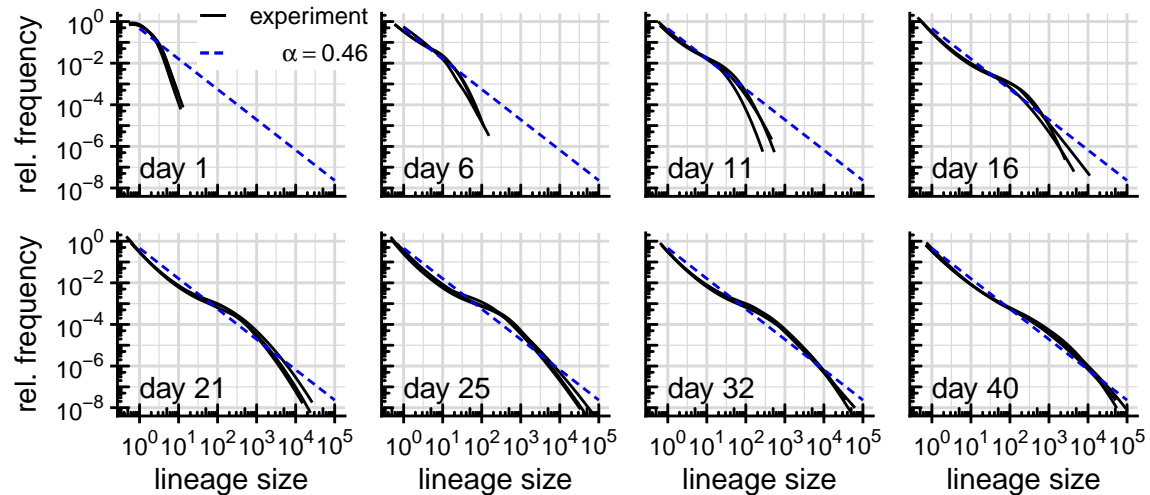

Supplement: S1 Fig — Relative frequencies of different lineage sizes on day 40 vs. Pareto power law with equality index α¯=0.46. (PDF) [file pcbi.1012054.s002.pdf]

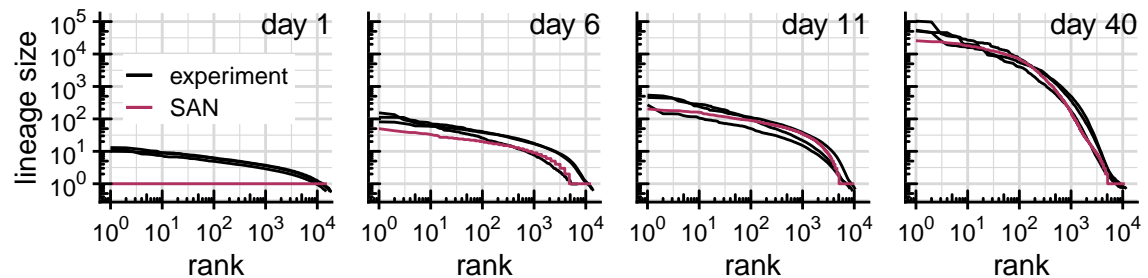

Supplement: S2 Fig — Rank-size distributions observed experimentally (black) and predicted by the SAN model (red). Other than in Fig 2D, the prediction shows the number of cells predicted by the model, not the number of cells observed through high-throughput sequencing. (PDF) [file pcbi.1012054.s003.pdf]

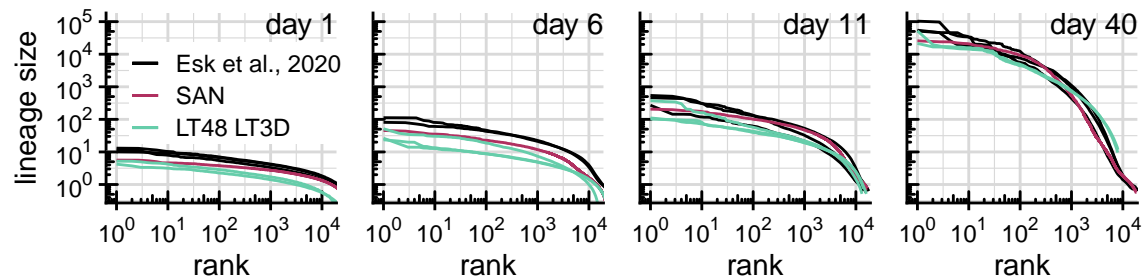

Supplement: S3 Fig — Replicate experiments based on the same organoid protocol show similar lineage size distributions as the data from Esk et al. (2020). Plots show three (days 6 and 11) respectively two (days 1 and 40) replicates; the replicates not shown were excluded due to sequencing quality issues. Ranks of the Esk et al. data and SAN model predictions were scaled to account for a 1.7-fold increase in the number of detected lineages in the replicate experiments. See S1 Supplemental Methods for experimental details. (PDF) [file pcbi.1012054.s004.pdf]

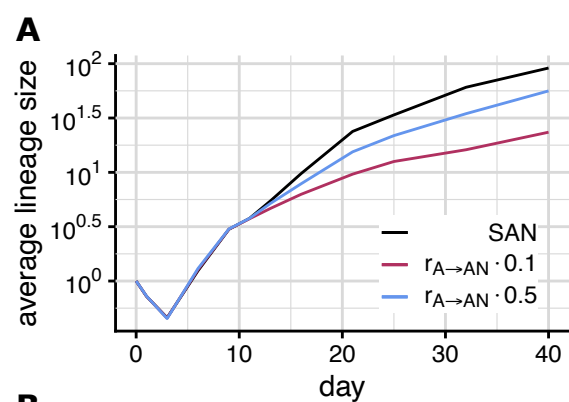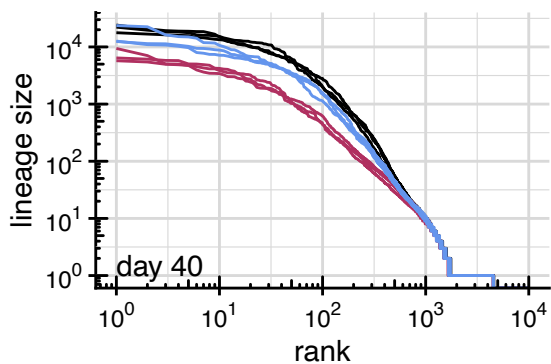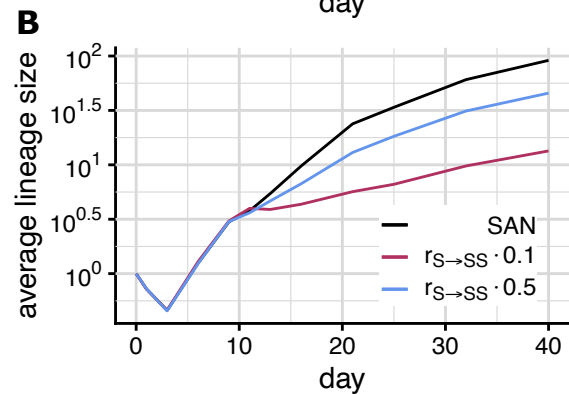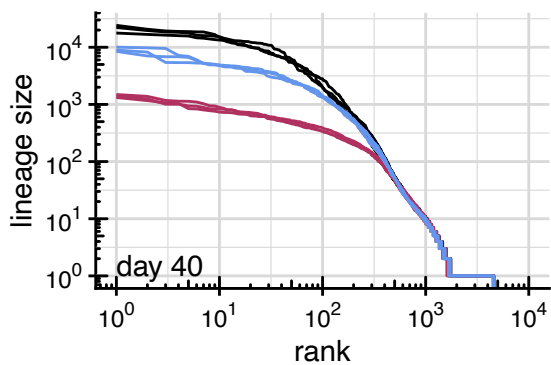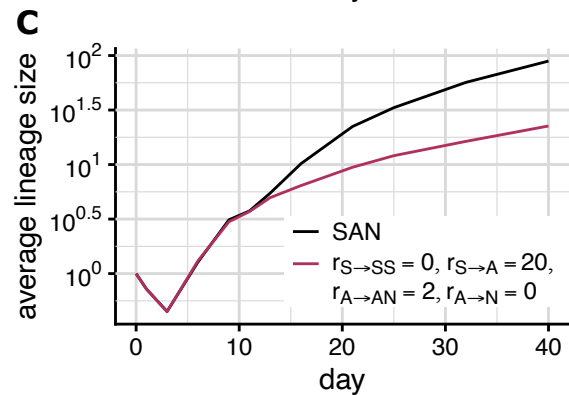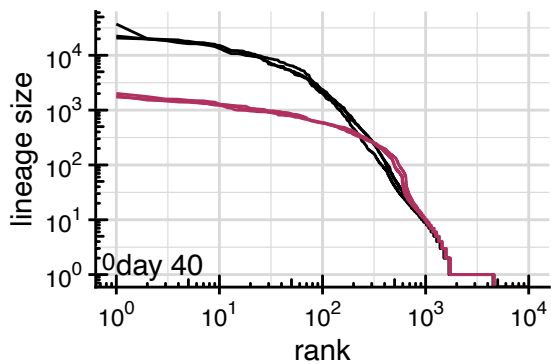

Supplement: S4 Fig — Rate of asymmetric division after day 11 reduced to one-half (blue) and one-tenth (black) of its original value. (B). Rate of symmetric division after day 11 reduced to one-half (blue) and one-tenth (black) of its original value. Rate of S -> A differentiations reduced accordingly to keep the net S-cell growth rate unchanged (C). Immediate differentiation of S-cells on day 11, no symmetric divisions. (PDF) [file pcbi.1012054.s005.pdf]

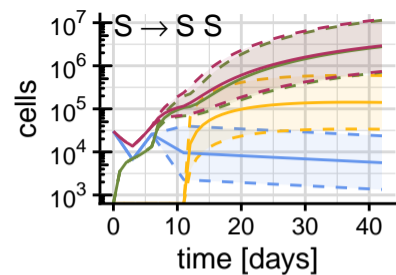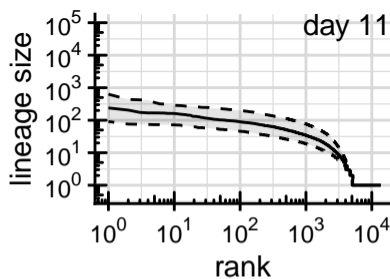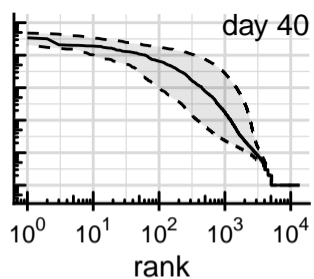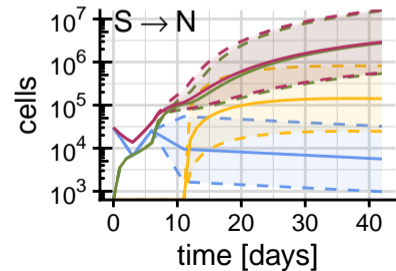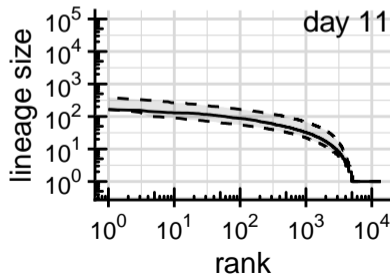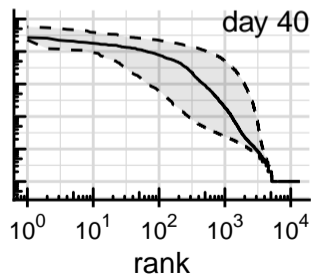

Supplement: S5 Fig — Model response to modified rates for days 11 to 40. MAP estimates (Table 1) are increased/decreased two standard deviations of the posterior (0.94 ± 0.28 for S → S S; 1.14 ± 0.35 for S → N, see also S6 Table). The leftmost plot shows the total organoid size (red), S-cells (blue), A-cells (yellow), and N-cells (green). The plots on the right show the lineage size distribution. Solid lines represent the unmodified model. Dashed lines represent the two modified models, the area in between is shaded. (PDF) [file pcbi.1012054.s006.pdf]

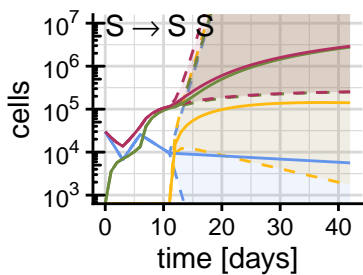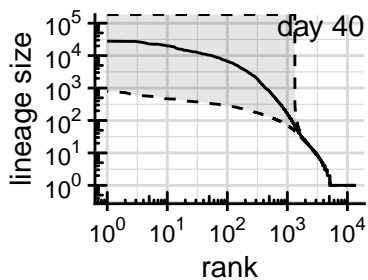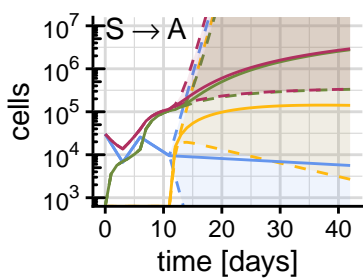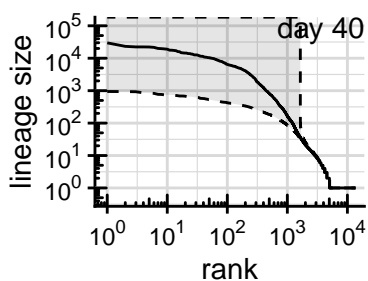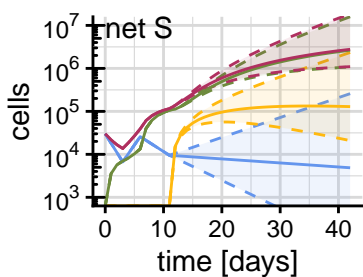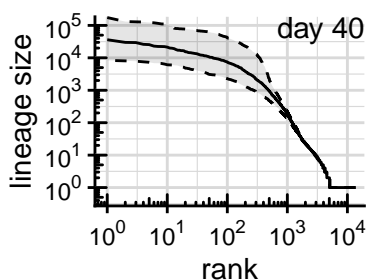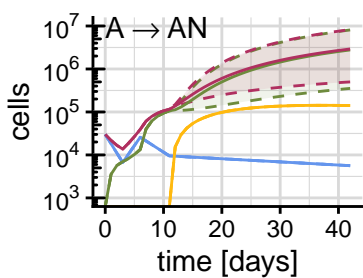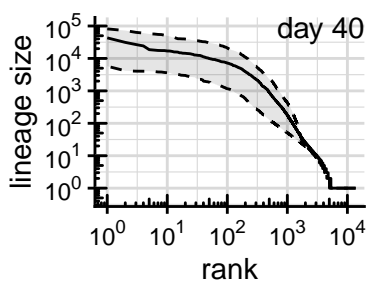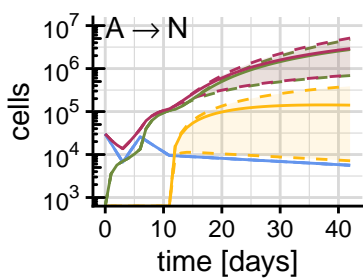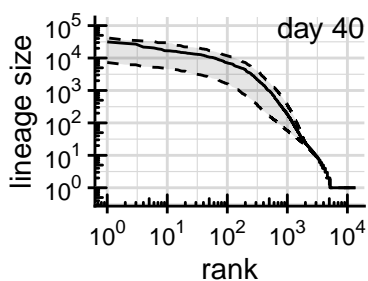

Supplement: S6 Fig — Model response to modified rates for days 11 to 40, including net S production S → S S minus S → A. MAP estimates (Table 1) are increased/decreased two standard deviations of the posterior (1.68 ± 1.04 for S → S S; 1.69 ± 1.18 for S → A; -0.022 ± 0.128 for net S; 0.71 ± 1.56 for A → AN; 0.072 ± 1.27 for A -> N; see also S6 Table). The leftmost plot shows the total organoid size (red), S-cells (blue), A-cells (yellow), and N-cells (green). The plots on the right show the lineage size distribution. Solid lines represent the unmodified model. Dashed lines represent the two modified models, the area in between is shaded. (PDF) [file pcbi.1012054.s007.pdf]
